# A Nondiagnostic 99mTc-PYP Scan with Absent Skeletal Uptake

**DOI:** 10.3390/diagnostics16121829

**Published:** 2026-06-12

**Authors:** Hiroyuki Tokue, Azusa Tokue, Yoshito Tsushima

**Affiliations:** Department of Diagnostic and Interventional Radiology, Gunma University Hospital, 3-39-22 Showa-Machi, Maebashi 371-8511, Gunma, Japan; azusana45@yahoo.co.jp (A.T.); yyoshitotushima@ymail.ne.jp (Y.T.)

**Keywords:** 99mTc-PYP, cardiac amyloidosis, free pertechnetate, radiopharmaceutical pitfall, SPECT/CT

## Abstract

99mTc-pyrophosphate (PYP) scintigraphy is widely used for the noninvasive evaluation of transthyretin cardiac amyloidosis. Although interpretation primarily focuses on myocardial uptake, confirmation of appropriate systemic radiotracer biodistribution is essential. We report a case in which an examination presumed to be 99mTc-PYP scintigraphy demonstrated free 99mTc-pertechnetate-like biodistribution. A 75-year-old woman with chronic kidney disease and conduction disturbance underwent 99mTc-PYP scintigraphy for suspected cardiac amyloidosis. The initial study, recorded as the administration of 740 MBq 99mTc-PYP, was imaged 3 h after injection. Planar imaging showed mild apparent activity over the cardiac region; however, SPECT/CT demonstrated no definite myocardial uptake. Instead, intense uptake was observed in the stomach and thyroid gland, with complete absence of skeletal activity. This distribution was inconsistent with correctly administered 99mTc-PYP and suggested free 99mTc-pertechnetate biodistribution, likely due to radiopharmaceutical preparation or administration error. A repeat 99mTc-PYP scan 1.5 months later showed expected skeletal uptake without gastric or thyroid activity and again demonstrated no myocardial uptake. The study was interpreted as negative for cardiac amyloidosis. Gastric and thyroid uptake with absent skeletal activity on presumed 99mTc-PYP scintigraphy should be considered nondiagnostic rather than negative.

**Figure 1:**
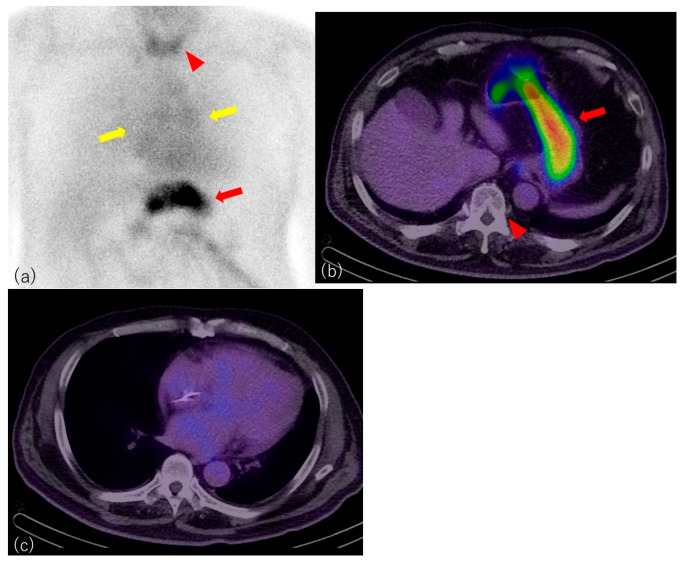
Initial examination presumed to be 99mTc-PYP scintigraphy. A 75-year-old woman with a history of chronic kidney disease, hypertension, and cerebral infarction was admitted for bradyarrhythmia. Electrocardiography showed advanced atrioventricular block and sick sinus syndrome, and a dual-chamber pacemaker was implanted. Serum troponin was elevated, but coronary angiography showed no significant coronary artery stenosis. Because cardiac amyloidosis was considered in the differential diagnosis, 99mTc-PYP scintigraphy was performed. Transthoracic echocardiography showed preserved left ventricular systolic function, with a left ventricular ejection fraction of approximately 55%. Left ventricular wall thickness was within the normal range, and no apical sparing pattern was identified. The patient had chronic kidney disease, with an estimated glomerular filtration rate of 29 mL/min/1.73 m^2^ and serum creatinine of 1.78 mg/dL (reference range, 0.46–0.79 mg/dL). No serum monoclonal protein or urinary Bence Jones protein was detected. The initial examination was presumed to be 99mTc-PYP scintigraphy. The administered activity was 740 MBq. Planar imaging and SPECT/CT were acquired 3 h after injection. Anterior and lateral planar images, axial SPECT images, and fused SPECT/CT images were available. In anterior planar imaging, slight uptake was observed in the cardiac region (yellow arrows), and uptake was seen in the thyroid gland (arrowhead) and stomach (arrow) (**a**). A fused SPECT/CT image at the upper abdominal level showed intense gastric uptake (arrow) and absence of uptake in the adjacent vertebral body (arrowhead), supporting abnormal systemic biodistribution (**b**). A fused SPECT/CT image at the cardiac level showed no definite myocardial uptake (**c**). Salivary gland uptake could not be assessed because the salivary glands were outside the imaging field of view. This distribution was markedly atypical for correctly administered 99mTc-PYP. The combination of gastric and thyroid activity with complete absence of skeletal uptake suggested free 99mTc-pertechnetate–like biodistribution. Therefore, the initial examination was considered nondiagnostic rather than negative for cardiac amyloidosis. After the abnormal biodistribution was recognized, the radiopharmaceutical preparation and administration process was reviewed. Routine radiochemical purity testing had not been performed before administration in this case, and no clear procedural irregularity was identified. No other patient underwent 99mTc-PYP scintigraphy using the same preparation on that day. A qualitative post hoc test of the residual PYP kit preparation was performed by the supplier using a silver nitrate reagent under acidic conditions with acetic acid. This test did not suggest gross labeling failure of the residual preparation. However, it did not provide a quantitative radiochemical purity value at the time of administration and could not confirm the composition of the injected syringe. Therefore, the exact mechanism could not be definitively established. Possible explanations included incomplete radiolabeling with a high fraction of free 99mTc-pertechnetate or inadvertent administration of free 99mTc-pertechnetate instead of the prepared 99mTc-PYP.

**Figure 2:**
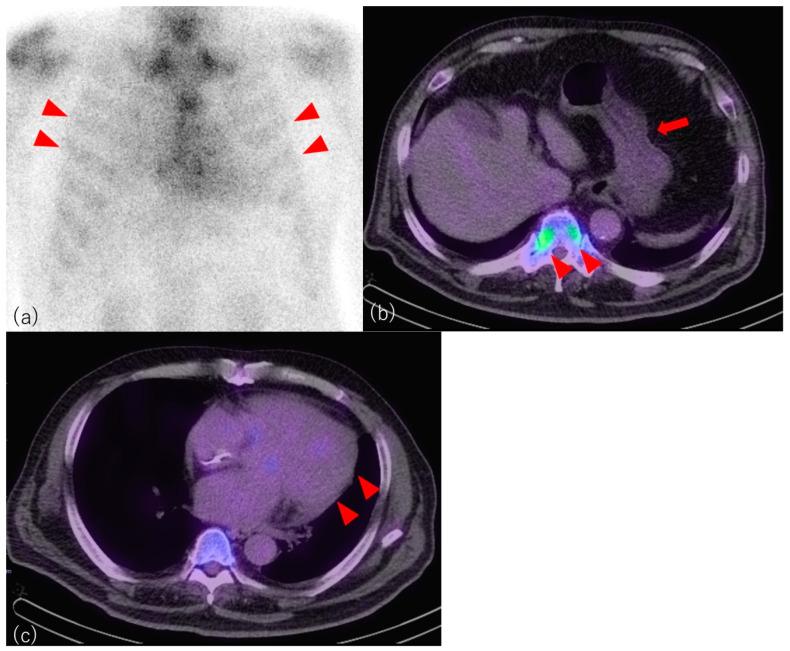
Repeat 99mTc-PYP scintigraphy. A repeat 99mTc-PYP scan was performed 1.5 months later. The administered activity was again 740 MBq, and planar imaging and SPECT/CT were acquired 3 h after injection under similar imaging conditions. The serum creatinine level at the time of the repeat examination was 1.75 mg/dL. In contrast to the initial examination, the repeat scan demonstrated expected skeletal uptake (arrowheads) on both planar images and SPECT/CT (**a**,**b**). The previously observed gastric (arrow) and thyroid activity had disappeared. Planar imaging showed apparent activity over the cardiac region, and the H/CL ratio was 1.18. However, SPECT/CT showed no definite myocardial uptake (arrowheads) (**c**). The repeat examination was therefore interpreted as negative for cardiac amyloidosis. Subsequent endomyocardial biopsy was performed from the left ventricular septum and inferior wall. Congo red staining showed no amyloid deposition. Transthyretin gene analysis revealed no pathogenic variant. Based on the repeat 99mTc-PYP findings, histopathological findings, and genetic analysis, cardiac amyloidosis was considered unlikely. Teaching points: Key imaging clues suggesting a nondiagnostic 99mTc-PYP study include prominent gastric or thyroid uptake, complete absence of skeletal activity, and discordance between apparent cardiac-region activity on planar imaging and absence of definite myocardial uptake on SPECT/CT. This case demonstrates an important imaging pitfall in 99mTc-PYP scintigraphy. Although the initial examination showed no definite myocardial uptake on SPECT/CT, the key finding was the abnormal systemic biodistribution: prominent gastric and thyroid uptake with complete absence of skeletal activity. This pattern is incompatible with a valid 99mTc-PYP study and suggests free 99mTc-pertechnetate–like biodistribution. In 99mTc-PYP scintigraphy for cardiac amyloidosis, visual grading is based on comparison between myocardial and rib uptake [1,2]. Therefore, the presence of expected skeletal activity is essential for interpretation. In a valid positive study, myocardial uptake is interpreted in relation to background skeletal, particularly rib, uptake. When skeletal uptake is absent, the standard visual grading system cannot be reliably applied. In the present case, slight apparent activity over the cardiac region on planar imaging could have led to misclassification as weakly positive, equivocal, or negative. However, SPECT/CT showed no definite myocardial uptake, and the complete absence of skeletal uptake indicated that the initial study was nondiagnostic. SPECT/CT was essential in this case because planar imaging alone showed mild apparent activity over the cardiac region and could have been misleading. Tomographic evaluation confirmed the absence of definite myocardial uptake and demonstrated abnormal systemic biodistribution, supporting classification of the initial examination as nondiagnostic. Free 99mTc-pertechnetate typically accumulates in the thyroid gland, salivary glands, gastric mucosa, and urinary tract, whereas skeletal uptake is not expected [3,4]. Gastric uptake on 99mTc-PYP imaging may also suggest hypercalcemia, hyperparathyroidism, or free pertechnetate contamination, and should prompt review of clinical, laboratory, and radiopharmaceutical factors [5]. The initial scan showed intense gastric uptake and thyroid uptake without skeletal activity, while repeat imaging under similar conditions showed expected skeletal uptake and disappearance of gastric and thyroid activity. This direct comparison supports the interpretation that the initial scan reflected abnormal radiopharmaceutical biodistribution rather than patient-specific physiology, consistent with prior reports emphasizing gastric uptake as a potential pitfall in 99mTc-PYP imaging [6]. Abnormal biodistribution in 99mTc-PYP imaging may result from several causes, including radiochemical purity failure, labeling inefficiency, inadvertent injection of an incorrect radiopharmaceutical, dose extravasation, and patient-related physiological factors. In clinical practice, these possibilities should be assessed by reviewing the distribution pattern, injection site, preparation and administration records, quality control procedures, and other examinations performed on the same day. In the present case, the combination of prominent gastric and thyroid uptake with complete absence of skeletal activity was most compatible with free 99mTc-pertechnetate–like biodistribution rather than dose extravasation or patient-specific physiology. The exact mechanism could not be definitively proven. Possible explanations include incomplete radiolabeling with a high fraction of free 99mTc-pertechnetate or inadvertent administration of free 99mTc-pertechnetate instead of the prepared 99mTc-PYP. A qualitative post hoc test of the residual PYP kit preparation using a silver nitrate reagent under acidic conditions did not suggest gross labeling failure, but this test did not confirm the composition of the injected syringe or provide a quantitative radiochemical purity value at the time of administration. Misinterpreting such a technically invalid study as negative or equivocal could delay repeat imaging and appropriate diagnostic evaluation for cardiac amyloidosis. In conclusion, this case emphasizes that prominent gastric and thyroid uptake with absent skeletal activity on a presumed 99mTc-PYP scan should not be interpreted as a negative cardiac amyloidosis study. Such findings should prompt review of radiopharmaceutical preparation and administration procedures, and the examination should be considered nondiagnostic.

## Data Availability

The original contributions presented in this study are included in the article. Further inquiries can be directed to the corresponding author.

## Abbreviations

The following abbreviations are used in this manuscript:

99mTc	Technetium-99m
PYP	Pyrophosphate
SPECT	Single-photon emission computed tomography
CT	Computed tomography
MBq	Megabecquerel
H/CL ratio	Heart-to-contralateral lung ratio
